# The Clinical View on *Streptococcus anginosus* Group – Opportunistic Pathogens Coming Out of Hiding

**DOI:** 10.3389/fmicb.2022.956677

**Published:** 2022-07-08

**Authors:** Magdalena Pilarczyk-Zurek, Izabela Sitkiewicz, Joanna Koziel

**Affiliations:** ^1^Department of Microbiology, Faculty of Biochemistry, Biophysics and Biotechnology, Jagiellonian University, Krakow, Poland; ^2^Center for Translational Medicine, Warsaw University of Life Sciences (SGGW), Warszawa, Poland

**Keywords:** *Streptococcus anginosus* group, opportunistic pathogens, bacteremia, abscesses, empyema, clinical infection, *Streptococcus milleri* group

## Abstract

Three distinct streptococcal species: *Streptococcus anginosus, Streptococcus intermedius*, and *Streptococcus constellatus*, belonging to the *Streptococcus anginosus* group (SAG), also known as *Streptococcus milleri* group, have been attracting clinicians and microbiologists, not only as oral commensals but also as opportunistic pathogens. For years they have been simply classified as so called viridans streptococci, and distinct species were not associated with particular clinical manifestations. Therefore, description of SAG members are clearly underrepresented in the literature, compared to other medically relevant streptococci. However, the increasing number of reports of life-threatening infections caused by SAG indicates their emerging pathogenicity. The improved clinical data generated with the application of modern molecular diagnostic techniques allow for precise identification of individual species belonging to SAG. This review summarizes clinical reports on SAG infections and systematizes data on the occurrence of individual species at the site of infection. We also discuss the issue of proper microbiological diagnostics, which is crucial for further clinical treatment.

## Introduction

The *Streptococcus group was described for milleri* the first time by Guthof in 1956, after being isolated from dental abscesses. The species name was chosen to honor arguably one of the most important practicing dentists, W. D. Miller, who spent his time in Robert Koch’s Laboratory to identify the germs responsible for tooth decay. Miller’s chemo-parasitic theory, together with the description of “gelatinous microbic plaques,” now commonly known as “dental plaque,” provided the key elements of our modern concept of the etiology of dental caries (He and Shi, 2009). Due to the lack of a single international nomenclature and the lack of established phenotypic markers, the *Anginosus* group of the genus *Streptococcus* was a subject of taxonomic confusion. The name “*Streptococcus milleri* group” has been used by European and Japanese microbiologists, including all group members, while North American microbiologists have been using terms such as *Streptococcus MG-intermedius* and *Streptococcus anginosus-constellatus*. SMG is still in use, but the term “*Streptococcus anginosus* group” (SAG) proposed by Kawamura is preferred (Jensen et al., 2013). SAG consists of three species designated *Streptococcus anginosus, Streptococcus constellatus*, and *Streptococcus intermedius*. Recent data suggest that the *Anginosus* group exhibits higher taxonomic heterogeneity, including the following: *S. anginosus* subsp. *anginosus* (SAA) and subsp. *whileyi, S. constellatus* subsp. *constellatus*, subsp. *pharyngis*, and subsp. *viborgensis*, and *S. intermedius*. More recently, based on multilocus sequence analysis (MLSA), the new subspecies of *S. anginosus* (*S. anginosus* genomosubsp. *vellorensis*) was advocated and the type of SAA strain was clustered within the *S. anginosus* gssp. *vellorensis* (Whiley, et al., 1999; Jensen et al., 2013; Tabata et al., 2019).

The members of SAG are frequently found within different oral structures and tissues. *S. intermedius* and *S. constellatus* are often associated with dental plaque and periodontal diseases (Sitkiewicz, 2018), while *S. anginosus* colonizes the mucosal membranes of the oral cavity (Asam and Spellerberg, 2014). All species can also colonize the throat, nasopharynx, gastrointestinal tract, and genitourinary tract, caused by spreading from the oral cavity (Neumayr et al., 2010).

Until the 1990s (Vandamme et al., 1998) diagnostic procedures for the identification of streptococci and the classification of these bacteria into the *S. anginosus* group were based on phenotypic differences examined using biochemical tests. Among them was the Voges–Proskauer test (*Mac Faddin, 1980) that is positive for the *S*. *anginosus* group and not by other β-hemolytic streptococcal species (Coykendall et al., 1987). *S. anginosus* bacteria are positive for arginine dihydrolase and esculin hydrolysis and negative for mannitol and sorbitol fermentation (Gray, 2005). Differentiation in the production of sugar hydrolyzing enzymes, sialidase, and hyaluronidase, was also used to identify SAG (Whiley, et al., 1990). Grinwis and co-workers presented a very interesting analysis of the validity of using various SAG identification tests, including Lancefield grouping, hemolysis, production of hyaluronidase, chondroitin sulfatase, DNase, proteases, hydrogen peroxide, and PCR for the detection of the intermedilysin gene (*ily*) (Grinwis et al., 2010). Due to the multitude of tests required for precise diagnosis, SAG isolates were rarely identified at the species level before the 2000s when advanced molecular biology tests were introduced for the common practice. Therefore, SAGs have been considered for many years a part of the commensal human microbiota, and their presence in polymicrobial infection samples was neglected (Asam and Spellerberg, 2014). For these reasons, for many years, SAG species were not considered a source of serious clinical infections.

## *Streptococcus anginosus* Group – Opportunistic Pathogens

The fast development of diagnostic methods based on molecular microbiology techniques revealed that SAG should be reclassified as opportunistic pathogens, as those species may cause invasive infections after entering sterile sites in the body (Clarridge et al., 2001).

### Bacteremia

The population study in 1989–2000 shows episodes of SAG bacteremia with an incidence of 0.93 per 100,000 population per year (Weightman et al., 2004). However, the epidemiological report from 2010 to 2017 of SAG-induced bloodstream infections presented an increase in annual incidence showing 3.7 cases per 100,000 inhabitants (Laupland et al., 2018). The increase in the prevalence index of SAG was clearly documented, although it remains elusive if it depends on the awareness of the clinician or the improvement of bacterial identification methods (**OPEN QUESTION 1**).

The systemic distribution of SAG leading to bacteremia is a consequence of the disruption of the mucosal barrier, which supports bacterial invasion into the underlying tissue (Bert et al., 1998).

The first evidence of bacteremia in association with gingivitis, periodontitis, and tooth brushing was reported by Forner et al. (2006). SAG can spread to the blood in people with oral infections, such as gingivitis and tooth abscesses (Terzi et al., 2016). Periodontitis is also a possible risk factor for the translocation of bacteria from the oral cavity into the bloodstream through ulcerated inflamed crevice, pocket epithelium, and adjacent gingival microcirculation (Dhotre et al., 2018). The authors observed a high similarity rate of the *S. anginosus* and *S. constellatus* isolates with distribution among subgingival plaque and blood samples. It is noteworthy that Lockhart et al. (2009) showed that even seemingly harmless gingival bleeding that occurs after brushing the tooth may be associated with an almost eightfold increase in the risk of bacteremia. As described above, the oral cavity can be a primary source of *S. anginosus* bacteriemia, but some authors indicate also the hepatobiliary system or the urinary tract (Suzuki et al., 2016). The others suggest esophagus and stomach as the source of SAG in cancer patients (Wu and Zheng, 2020), especially in patients with gastric tumors (Liu et al., 2019). Examples of SAG-related bacteremia are described in Table 1.

**TABLE 1:** *Streptococcus anginosus* group-related bacteremia.

BACTEREMIA	Total number of:		Description of the study	References
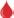				
SAG species	Isolates	Patients		
*S. anginosus*	21	122	Blood was second to abscess fluid as the most common sample from which the SAG was recovered.	Clarridge et al., 2001
*S. constellatus*	7			
*S. intermedius*	5			
*S. anginosus*	12	18	*S. anginosus* was particularly prominent among the blood and also urogenital, and perirectal abscess.	Flynn and Ruoff, 1995
*S. constellatus*	3			
*S. intermedius*	3			
*S. anginosus*	15	19	SAG bacteremia occurred about one-eighth as often as *Staphylococcus aureus* bacteremia and one-third as often as *Streptococcus pneumoniae, Klebsiella* species, or *Pseudomonas aeruginosa* bacteremia.	Jacobs et al., 1995
*S. constellatus*	2			
*S. intermedius*	2			
*S. anginosus*	19	245	The 21 bacteremic cases represented secondary invasion from purulent foci in various systems.	Siegman-Igra et al., 2012
*S. constellatus*	8			
*S. intermedius*	1			
*S. anginosus*	28	51	Forty-one patients (80.4%) had a single blood culture positive for SAG.	Bert et al., 1998
*S. constellatus*	22			
*S. intermedius*	1			
*S. anginosus*	18	105	More than half of the cases had one or more underlying comorbidities, diabetes, malignancies, and chronic kidney disease.	Al Majid et al., 2020

Among studies on SAG-induced mortality we can find the 3-year study (1990–1992), which showed that among examined 19 cases of SAG-associated bacteremia 5 patients died, pointing 26.3% of the mortality (Jacobs et al., 1994). On the other hand, another study conducted from 2015 to 2017 revealed only 6% overall mortality. The most recently published meta-analysis of 101 case reports published between 1996 and 2019 indicates that mortality rates among patients with SAG-associated bacteremia range between 10 and 16% (Issa et al., 2020).

### Diseases Caused by *Streptococcus anginosus* Group

The latest clinical data indicate the presence of life-threatening infections induced by SAG, as reflected in the formation of various types of empyemas and abscesses. They are the most commonly isolated aerobes from peritonsillar abscesses next to the group A streptococci (GAS) (Galioto, 2017). A retrospective cohort study conducted between 2009 and 2015 showed that SAG-causing pyogenic infections were identified among 160/263 patients (60%) and included intraabdominal abscesses or peritonitis (69 patients, 43.1%), skin/soft tissue abscesses, arthritis or osteomyelitis (44 patients, 28.1%), empyema or lung abscesses (32 patients including one with empyema and intraabdominal infection, 20%) and intracranial abscesses (15 patients, 9.4%) (Kobo et al., 2017).

In particular, clinical data indicate the different rate of prevalence among SAG species, as well as tissue specificity. The retrospective analysis (2014–2019) of the 463 samples revealed 254 of *S. anginosus* (54.86%), 173 of *S. constellatus* (37.37%), and 36 of *S. intermedius* (7.77%) (Jiang et al., 2020). *S. anginosus* is more commonly isolated from gastrointestinal and genitourinary tract infections, *S. constellatus* has a propensity for the respiratory tract, while *S. intermedius* is responsible for most head and neck infections and infections of the central nervous system (CNS) (Clarridge et al., 2001). Similar data were recorded in studies carried out with 3–10 years old children. The authors identified *S. anginosus* in more than 70% of genitourinary tract infections, *S. constellatus* in skin and soft tissue diseases, and *S. intermedius* in head and neck diseases (Whiley, et al., 1992; Furuichi and Horikoshi, 2018).

Moreover, *S. intermedius* has the tendency to form abscesses and infection of deep tissues and is more likely to cause supportive, non-bacteremic infections, which require surgical intervention. On the contrary, *S. anginosus* and *S. constellatus* were associated with bacteremia, but have a lower incidence of pyogenic infection (Wu and Zheng, 2020).

In the following, we describe the clinical data showing different tissue-specific SAG infection with their pathological consequences. Detailed information is presented in Table 2.

**TABLE 2:** Co-morbidity of SAG with different disorders.

Body part	Disorders	*Streptococcus* species	Total number of		Description of the study	References
			Isolates	Patients		
Head and neck	Intracranial abscess	*S. anginosus*	1	1	Multiple intracranial abscesses in a previously well individual	Kirkman et al., 2012
	Brain abscess 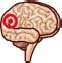	*S. intermedius*	19	39	Diagnosis was secured in 39 patients, among which the majority were SAG (69%) with a predominance of *S. intermedius* (70%).	Darlow et al., 2020
		*S. intermedius*	2	2	Predisposing condition was a paranasal sinusitis of the frontal and ethmoidal sinuses	Yamamoto et al., 1999
		*S. anginosus*	1	1	Case represents a frontal lobe abscess caused by contiguous spread of *S. anginosus* from a frontal sinus infection	Esplin et al., 2017
	Peritonsillar abscess 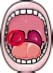	*S. constellatus*	13	65	67 aerobic isolates from peritonsillar abscess 55 were classified as *Streptococcus* spp. with the SAG as the most common type (20 isolates).	Hidaka et al., 2011
		*S. intermedius*	7			
	Orofacial abscesses	*S. intermedius*	19	198	The strains of SAG were encountered most frequently (81% of infections) as part of a mixed growth.	Wilson, et al., 1995
		*S. constellatus*	16			
		*S. anginosus*	8			
		*S. anginosus*	1	6	Septic cavernous sinus thrombosis and orbital cellulitis	Branson et al., 2019
	Lemierre’s syndrome	*S. anginosus*	1	1	Case report	Santos et al., 2020

Pulmonary disorders	Lung abscess	*S. constellatus*	21	72	Species belonging to the SAG accounted for the majority 68% of isolates	Jerng et al., 1997
		*S. intermedius*	17			
	Pleural empyema 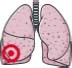	*S. intermedius*	16	30	*S. intermedius* was significantly more common in patients with lung abscess with pleural effusion	Noguchi et al., 2015
		*S. constellatus*	11			
		*S. anginosus*	3			
	Pneumonia	*S. constellatus*	16	31	Empyema was observed in patients with multiple bacteria	Hirai et al., 2016
		*S. anginosus*	13			
		*S. intermedius*	2			
		*S. intermedius*	1	1	Immunocompetent patient	Hannoodi et al., 2016

Cardiovascular diseases	Pericarditis 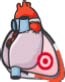	*S. anginosus*	4	4 case reports	Pneumopericardium secondary to gastropericardial fistula, purulent pericardial effusion secondary to transdiaphragmatic rupture of pyogenic liver abscess	Maves et al., 2017; Cai, 2020; Prateepchaiboon et al., 2020; Ono et al., 2021
						
		*S. intermedius*	3	3 case reports	The first reported case of purulent pericarditis with *S. intermedius* in a child, patient with the history of bronchiectasis and pneumonia	Presnell et al., 2014; Tigen et al., 2015; Khan et al., 2018
	Endocarditis	*S. anginosus*	6	377	All SAG isolates were identified as *S. anginosus*	Woo et al., 2004
						
	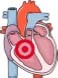	*S. constellatus*	11	11	Infection tends to be complicated with a high frequency of septic embolization	Yoshino et al., 2013

Liver disorders	Liver abscess 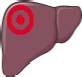	*S. intermedius*	11	11 case reports	Bacteremia and liver abscess following a routine dental infections, infection associated with gastrointestinal stromal tumor, diverticulitis of the colon, adenocarcinoma of the colon, acute cholangitis sump syndrome, chronic granulomatous disease	Hiura et al., 2000; Millichap et al., 2005; Wagner et al., 2006; Neumayr et al., 2010; Falcone et al., 2012; Livingston and Perez-Colon, 2014; Benou et al., 2016; Mayor et al., 2016; Parthvi et al., 2017; Reddy et al., 2018; Hanna et al., 2020
						
		*S. constellatus*	5	5 case reports	Diverticulitis of the sigmoid colon and subsequent hematogenous spread of bacteria, infection associated with gastric adenocarcinoma	Akuzawa et al., 2017; Dsouza et al., 2019; Wong, et al., 2019; Chrastek et al., 2020; Navarrete et al., 2020

#### Head and Neck

*Streptococcus anginosus* group have a potential to extend in the head and neck, therefore their presence requires special attention considering the proximity of vital organs as CNS (Han and Kerschner, 2001).

**Chronic maxillary sinusitis**, which is often secondary to dental infection, is quite common among head and neck diseases. The main complication of sinusitis is the local spread of bacterial infection, causing periorbital or orbital cellulitis, cavernous sinus thrombosis, epidural, or brain abscess. Hutchin and co-workers emphasize the correlation between the occurrence of *S. anginosus*, sinusitis, and sinogenic subdural empyema. The authors proposed that the growth of microaerophilic SAG is favored as oxygen content decreases as a result of closure of the sinus ostia associated with mucosal edema and inflammation (Hutchin et al., 1999).

**Peritonsillar abscess** is an acute pharyngeal infection most common among adolescents and young adults and is considered a complication of acute tonsillitis or peritonsillar cellulitis (Passy, 1994). Interestingly, it was suggested that accelerated inflammation is an effect of coexistence of the SAG with other anaerobes, such as *Fusobacterium necrophorum* and *Prevotella melaninogenica*, but also *Prevotella intermedia, Peptostreptococcus micros, Fusobacterium nucleatum*, and *Actinomyces odontolyticus* (Jousimies-Somer et al., 1993; Hidaka et al., 2011). As the literature lists groups of bacteria associated with peritonsillar abscess, the open question is whether there is a profile of coexistence among the identified groups of bacteria (**OPEN QUESTION 2**).

An **abscess of the brain** can result from direct extension of cranial infections (e.g., osteomyelitis, sinusitis, and subdural empyema), penetrating head wounds (including neurosurgical procedures) or hematogenous spread (e.g., in bacterial endocarditis). It was shown that SAGs are significantly more likely than other bacteria to cause severe intracranial complications and neurological disorders in pediatric patients with rhinosinusitis. In the 50 cases identified, *S. anginosus* groups was the most commonly implicated bacterial pathogen in 14 (28%) (Deutschmann et al., 2013). In a retrospective analysis of cases of brain abscesses, SAGs were the most commonly isolated microorganisms in brain abscesses (Carpenter et al., 2007). The clinical association of *S. intermedius* with the tendency to form head and neck abscesses has long been recognized. Among the reported cases, there is also a case of a highly disseminated purulent infection caused by *S. intermedius* involving the brain, lungs, liver, and pleural cavity (Giuliano et al., 2012).

As described above, head and neck infections are caused by SAG, however, the routes of bacterial spread are poorly explored (**OPEN QUESTION 3**). Among the routes considered for brain infection are contiguous infection, hematogenous spread, direct implant, trauma or neurosurgery, and peripheral nerves (Kragha, 2016).

#### Pulmonary Disorders

Another possibility of SAG expansion is aspiration, which can lead to the development of pulmonary diseases, e.g., pneumonia, lung abscess, and pleural empyema. Several mechanisms have been suggested for SAG-causing thoracic infections, including aspiration of oral secretion, direct implantation by trauma or surgery, extension by contiguity, and hematogenous dissemination (Kobashi et al., 2008). The dissemination of SAG to the lungs from the oral cavity, leading to abscess, was demonstrated by the study by Mukae et al. (2016) The authors examined bronchoalveolar lavage fluid samples. Among the bacteria most frequently detected were SAG (15.3%), followed by *Fusobacterium* spp. (23.7%) (Mukae et al., 2016). The retrospective analysis carried out by Noguchi et al. (2015) revealed that among a total of 30 patients diagnosed with respiratory infection (pneumonia, lung abscess, and bacterial pleurisy only), *S. intermedius, S. constellatus*, and *S. anginosus* were identified in 16 (53.3%), 11 (36.7%), and 3 (10.0%) patients. Other retrospective studies describing the bacteriology and clinical features of empyema thoracis and lung abscess showed that among the 76 isolated strains of viridans streptococci, the most common were *S. constellatus* (21 strains), *S. intermedius* (17), and *Streptococcus sanguis* (10) (Jerng et al., 1997). Moreover, there is an increase in the number of case reports that indicate *S. constellatus* as a pulmonary pathogen (Morinaga et al., 2013; Elhussein and Hutchison, 2014; Price et al., 2015; Zhang et al., 2020; Vulisha et al., 2021). Taken together, the role of SAG in the pathogenesis of lung diseases is indisputable, with the leading role of *S. constellatus* and *S. intermedius*.

#### Cardiovascular Diseases

Bacterial pericarditis usually occurs as a secondary infection induced by contiguous spread from the surrounding intrathoracic area, including extension from the pulmonary, myocardial, and subdiaphragmatic site. It is also a consequence of hematogenous dissemination from distant organs (Masood et al., 2016). Purulent pericarditis can be caused by SAG due to dental problems (Li et al., 2013). Secondary pericarditis caused by *S. anginosus* has been reported four times since 2000, twice as a complication after *S. intermedius* infections. The primary purulent pericarditis is extremely rare, including one case in which *S. anginosus* culture grew confirmed and another caused by S. *intermedius* (Khan et al., 2018; Beom et al., 2021).

Among the etiological agents of infectious endocarditis, streptococci range from 60 to 80%. A detailed microbiological examination revealed that *S. anginosus* rarely induces infective endocarditis (0.1%) (Stryjewski et al., 2015). During a 5-year period (1997–2002), 6 cases of SAG endocarditis were documented (Woo et al., 2004). Infective endocarditis due to *S. constellatus* revealed non-specific initial symptoms, especially coughing, and complications of abscess formation and septic embolization. The choice of antibiotic agent for the treatment of infective endocarditis due to *S. constellatus* should receive special attention, because penicillin resistant strains have been documented in some cases (Yoshino et al., 2013).

#### Liver Abscesses

*Streptococcus anginosus* group have been found to cause infections within the abdominal cavity including liver abscesses, which are usually identified as polymicrobial. Microorganisms associated with liver abscesses include most commonly Gram-negative enteric bacteria (*Escherichia coli, Klebsiella pneumoniae, Pseudomonas* sp., and *Proteus* sp.), Gram-positive aerobes (SAG, *Enterococcus* sp., *Staphylococcus aureus, Staphylococcus epidermidis*, and *Streptococcus* sp.), anaerobic organisms (*Bacteroides* sp. and *Fusobacterium), Actinomyces, Candida albicans, Salmonella typhi, Brucella melitensis*, or other protozoa (*Entamoeba histolytica* and *Echinococcus granulosus*) (Ioannou et al., 2016). The study of Meddings et al. (2010) shows that among culture-positive patients with pyogenic liver abscess, the most common organisms isolated from pus were *Streptococcus* species (29.5%). A retrospective study of patients with pyogenic liver abscess presenting that the most common causative pathogens were SAG 25%, *K. pneumoniae* 21%, and *E. coli* 16%, respectively (Pang et al., 2011). From 2000, few clinical cases of liver abscess due to monospecies infection with *S. intermedius* were presented. Among the recent reports we can also find clinical studies describing multiple liver abscesses of mixed origin, with the presence of *S. constellatus*. The results of the investigation suggested that diverticulitis of the sigmoid colon and subsequent hematogenous spread of *S. constellatus* may have led to the formation of liver abscesses and bacteremia.

The data cited above indicate the dominance of *S. constellatus* and *S. intermedius* in the pathogenesis of liver abscess. Notably, it was revealed that these species replicate more rapidly in the presence of other species, including *Eikenella corrodens* (Young et al., 1996). A clinical report that studied liver abscess in patients showed that *S. constellatus* was the most common organism co-isolated with *E. corrodens* in liver abscess (*n* = 3), pleural effusion (*n* = 2), blood (*n* = 1), and brain abscess (*n* = 1). As streptococci and *E. corrodens* are members of endogenous flora in the mouth and upper respiratory tract, their coexistence in the liver abscess suggests the oral cavity as the common source of infection (Sheng et al., 2001). Moreover, it also suggests that the coexistence of SAG with other microorganisms could bidirectionally support the increase in their pathogenicity (**OPEN QUESTION 4**).

## The Risk Factors for *Streptococcus anginosus* Group Infection

The incidence of SAG infection development increased with the occurrence of systemic diseases. The data indicate that clinically relevant predisposing factors were identified in 65% of the SAG infections studied. The most common were related to gastrointestinal tract pathology and included diverticular disease, cancer, biliary tract procedures and intestinal perforation, cirrhosis, Crohn’s disease, and variceal esophageal bleeding (Takeuchi et al., 2017). The significant common risk factors for blood infection were bariatric surgery, appendicular abscess, and appendectomy (Al Majid et al., 2020). The study by Jiang et al. (2020) describes risk factors for patients infected with SAG. A total of 210 of the 463 patients had major underlying diseases. Among them, 63 (30%) with solid tumors, 6 (2.86%) with hematologic malignancies, 70 (33.33%) with type 2 diabetes mellitus, 23 (10.95%) with diseases of the central nervous system (cerebral infarction, cerebral hemorrhage, brain trauma, myasthenia gravis, or Parkinson’s disease), 20 (9.52%) with chronic kidney failure, 12 (5.71%) with chronic respiratory disease, 6 (2.86%) with viral hepatitis, and 10 (4.77%) with connective tissue disease (Jiang et al., 2020).

*Streptococcus anginosus* group, as other microorganisms, have been shown to pass through the intestinal barrier and translocate to extra intestinal organs, thus increased intestinal permeability could be a plausible route of infection (Purohit et al., 2008). The presence of a cancerous lesion provides an insult to the normal colonic mucosa, allowing pathogens to invade the circulation. Therefore, colorectal carcinoma or rectal adenocarcinoma is a risk factor for SAG infection (Tzur et al., 2003; Lin et al., 2008; Masood et al., 2016). In addition, systemic *S. anginosus* infections have also been reported in patients with esophageal, gastric, and oral cancer (Sasaki et al., 2005; Masood et al., 2016). Another convincing report comes from the study of neutropenia patients. The authors revealed that the source of *S. anginosus* group infections is usually the gastrointestinal tract (GI). The study by Wenzler et al. (2015) found 18 cases (53% of 34 patients) with a gastrointestinal source of bacteremia; therefore, sepsis could have originated from an unidentified source of the GI tract. Siegman-Igra et al. (2012) reported 215 cases of SAG infections, the most common sources being the liver and other intraabdominal abscesses. In addition to cancer, there are many conditions that lead to a state of reduced immunity, which could predispose to SAG infection. Interesting data comes from the clinical case study of Reddy et al. (2018) who identified risk factors leading to the development of abscesses with the participation of *S. intermedius*. Among them, the authors revealed chronic diseases (56% of patients with type 2 diabetes, high blood pressure, AIDS, or cancer), unhealthy diet, alcohol, drug consumption, and smoking (33% patients) and surgical intervention, including dental work or laparoscopic cholecystectomy (11% cases) (Reddy et al., 2018). The age seems also to play a role in the course of disease as significantly higher mortality was reported in patients with SAG infections over 65 years of age, which was also associated with polymicrobial infections and immunosuppression (Al Majid et al., 2020). The list of risk factors is summarized in Table 3.

**TABLE 3:** Risk factors of infection with the *Streptococcus anginosus* group (SAG).

Systemic risk factors	Local risk factors	Environmental risk factors
Inflammation and ulceration of the gingival tissues	dental manipulation	Smoking
Periodontal disease (periodontitis, gingivitis)	Myocardial infarction	Heavy alcohol consumption
Solid tumors	Surgical interventions	Use of drugs
Hematologic malignancies		
Type 2 diabetes mellitus		
Central Nerve System Diseases (cerebral infarction, cerebral hemorrhage, brain trauma, myasthenia gravis, and Parkinson’s disease)		
Chronic kidney failure		
Chronic respiratory disease		
Heart failure		
Liver diseases		
Dementia		
Viral hepatitis		
Peptic ulcer disease		
		

## Identification Methods

The role of SAG in the pathogenesis of infectious diseases has been poorly described. This is a consequence of the difficulties in categorizing and identifying these microorganisms. Identification of *S. anginosus* group based on Lancefield groups is not applicable, as all species show high antigenic heterogeneity and include cross-reactive strains that react with Lancefield groups A, C, F, and G antisera (Grinwis et al., 2010). Therefore, commercial systems were developed, including: API 20 Strep and API Rapid 32 ID Strep (bioMérieux, France), the Fluo-Card Milleri kit (Flynn and Ruoff, 1995), or The VITEK^®^ 2 system. Despite being simple and quick tests, these methods provide reliable identification at the group level, not species (Teles et al., 2011). There is no gold standard for the identification of SAG. Cultivation methods and the available biochemical test are not sufficient for precise identification of the species, therefore it is necessary to use molecular techniques (Wenzler et al., 2015; Kragha, 2016). Among them, two 16S rRNA real-time PCR assays were developed. The 16s_SA assay is specific for *S. anginosus* (100%), while the 16s_SCI assay is specific for *S. constellatus* and *S. intermedius* (100%). These assays can detect <10 genome equivalents in pure culture and >10^4^ genome equivalents in sputum samples, making this a great tool for assessing the presence of SAG in complex polymicrobial samples. Note that it is not possible to distinguish between *S. constellatus* and *S. intermedius* by this method (Olson et al., 2010).

Another technique was designed on the basis of known streptococcal *gro*ESL sequences. The authors designed a pair of primers to differentiate the members of the SAG from other members of the *viridans* group streptococci (Liu et al., 2006). In order to further differentiate the species (*S. anginosus* and *S. constellatus*) among the anginosus group, amplification products were subsequently digested with the restriction enzymes. Therefore, the PCR restriction fragment length polymorphism (RFLP) could be used as a confirmatory test for the identification of the Anginosus group and differentiation between *S. anginosus* and *S. constellatus*. *S. intermedius* still remain unrecognized (Liu et al., 2006). Poyart et al. (1998) described the method to identify clinical streptococci isolates at the species level by determining the positions of their *sodA* gene that encodes a manganese-dependent enzyme.

To correctly identify *S. intermedius*, an accurate PCR identification system with the *ily* gene as a specific marker must be used. The amplification of fragment of the *ily* gene and its 3′-flanking region is specific for *S. intermedius* strains among all other streptococcal species (Teles et al., 2011). In recent studies, authors underline the promising potential of real-time PCR, rapid microarray assay, as well as mass spectrometry (MALDI-TOF-MS) in discriminating between the individual species of the *S. anginosus* group (Desar et al., 2008; Reißmann et al., 2010; Wenzler et al., 2015).

Accurate results were obtained for *S. anginosus* and *S. constellatus* which were identified at the species level by MALDI-TOF-MS (Woods et al., 2014). In addition, the developers of the MALDI Biotyper 3.1 software expand its database to cover, *inter alia, S. intermedius* strains to identify bacterial strains more accurately (Wei et al., 2021). The most promising diagnostic tool that can be used to identify and precisely distinguish between *viridans* streptococci is next generation sequencing (NGS). The low cost of sequencing, below cents per base, allows us to apply NGS for direct sequencing of bacteria from clinical samples. It is very useful to detect bacteria causing mixed infections, common in SAG infections. It speeds up the diagnostic process because the initial diagnosis does not need the growth of slowly growing SAG. Moreover, recent reports show that NGS combined with machine learning can be also used to predict MIC values (Nguyen et al., 2018). The disadvantage of NGS as widespread diagnostic tool is current lack of user friendly software that allow sequence based detection. However, due to rapid development of the technology it may change in the near future (Kozińska et al., 2019).

Jensen et al. (2013) presents re-examine the taxonomy SAG to seven distinct clusters based on MLSA combined with 16S rRNA gene sequence and phenotypic analyses. Isolates from newly described groups 3, 4, and 7 were of genetically homogeneous nature and contained β-hemolytic strains belonging to the Lancefield group C. The strains were mostly isolated from throat infections. The taxonomic presented at work will allow for a correct diagnosis which will facilitate a full understanding of the clinical significance (Jensen et al., 2013).

It is clear that the application of the molecular biology techniques has allowed for a more precise identification of SAG, thereby eliminating ambiguous or erroneous results that may have been unavoidable in the past (**OPEN QUESTION 5**).

## Conclusion

The precise identification of *viridans* streptococci at the species level is of great clinical importance. Every year, there are more and more reports of life-threatening infections with SAG. The surprise of clinicians with the identification of SAG streptococci as the etiological factor of the cases studied shows how much more remains to be learned about clinical pictures and diagnostic best practices. The long list of risk factors predisposing to the development of serious infections induced by SAG pointing them as community acquired pathogens. Increased susceptibility of people and repeated documented indications of the clear participation of SAG in serious infections show that SAG appear as emerging opportunistic pathogens.

### Open Questions

1. What is the real prevalence rate of SAG infections and the mortality rate?
2. Is there a profile of coexistence among the identified groups of bacteria?
3. Do SAGs have the ability to spread through the neural pathway?
4. Do SAGs act as independent pathogens, become harmful in relation to other microbes, or may support the pathogenicity of other microbes?
5. Is there a need to design a unified diagnostic scheme that leads to the precise identification of individual SAG species in clinical materials?

## Author Contributions

MP-Z, IS, and JK created conception and design of the study and wrote sections of the manuscript. All authors contributed to manuscript revision, read, and approved the submitted version.

## Conflict of Interest

The authors declare that the research was conducted in the absence of any commercial or financial relationships that could be construed as a potential conflict of interest.

## Publisher’s Note

All claims expressed in this article are solely those of the authors and do not necessarily represent those of their affiliated organizations, or those of the publisher, the editors and the reviewers. Any product that may be evaluated in this article, or claim that may be made by its manufacturer, is not guaranteed or endorsed by the publisher.
